# 肺癌细胞中GTSE1与细胞周期的关系及潜在调控机制

**DOI:** 10.3779/j.issn.1009-3419.2024.106.13

**Published:** 2024-06-20

**Authors:** Chuanlin WANG, Jiali XU, Mingzhu LIU, Jiayu LIU, Yunchao HUANG, Lan ZHOU

**Affiliations:** ^1^650118 昆明，昆明医科大学第三附属医院，云南省肿瘤医院临床营养科（王传林，徐佳丽，刘明珠，周岚）; ^1^Department of Clinical Nutrition, The Third Affiliated Hospital of Kunming Medical University, Yunnan Tumor Hospital, Kunming 650118, China; ^2^230032 合肥，安徽医科大学公共卫生学院卫生毒理学系（刘佳宇）; ^2^Department of Health Toxicology, School of Public Health, Anhui Medical University, Hefei 230032, China; ^3^650118 昆明，昆明医科大学第三附属医院，云南省肿瘤医院胸外科（黄云超）; ^3^Department of Chest Surgery, The Third Affiliated Hospital of Kunming Medical University, Yunnan Tumor Hospital, Kunming 650118, China

**Keywords:** 肺肿瘤, 细胞周期, GTSE1, 细胞周期调节因子, Lung neoplasms, Cell cycle, GTSE1, Cell cycle regulator

## Abstract

细胞周期的调控对于维持细胞正常功能至关重要，尤其在肺癌等疾病的发展中。细胞周期由四个主要阶段组成（G_1_、S、G_2_和M期），这些阶段通过一系列精确的分子事件来确保细胞正确增殖和分裂。在肺癌细胞中，细胞周期失调可导致癌细胞无序增殖和侵袭能力增强。G_2_和S期表达-1（G_2_ and S-phase expressed 1, GTSE1）是一种在细胞质中发现的调节蛋白，它在多种癌细胞的细胞周期分布中起着关键作用，并参与了细胞增殖和凋亡等过程。GTSE1通过与细胞周期蛋白依赖激酶抑制因子1A（cyclin-dependent kinase inhibitor 1A, p21）的相互作用，维持p21的稳定性，进而抑制细胞周期蛋白依赖性激酶1/2（cyclin-dependent kinase 1/2, CDK1/2）的活性，影响细胞周期的进程。此外，GTSE1还参与肿瘤蛋白53（tumor protein 53, p53）信号通路的调控。在双微体同源基因2（mouse double minute 2 homolog, MDM2）的协助下，GTSE1能够将p53从细胞核转运至胞质，并促进其泛素化降解，从而影响细胞周期和细胞死亡相关信号通路。本文综述了GTSE1在肺癌细胞中的表达情况和对肺癌的影响，以及其参与细胞周期调控的潜在机制。

## Abstract (en)

The regulation of the cell cycle is essential for maintaining normal cellular function, especially in the development of diseases such as lung cancer. The cell cycle consists of four major phases (G_1_, S, G_2_ and M phases), which are characterized by a series of precise molecular events to ensure proper cell proliferation and division. In lung cancer cells, cell cycle dysregulation can lead to disordered proliferation and increased invasiveness of cancer cells. G_2_ and S-phase expressed 1 (GTSE1) is a regulatory protein found in the cytoplasm of the cell, which plays a key role in the cell cycle distribution of a wide range of cancer cells and is involved in life processes such as cell proliferation and apoptosis. GTSE1 affects cell cycle progression by interacting with cyclin-dependent kinase inhibitor 1A (p21) and maintaining the stability of p21, which in turn inhibits the activity of cyclin-dependent kinase 1/2 (CDK1/2). In addition, GTSE1 is also involved in the regulation of tumor protein 53 (p53) signaling pathway. With the assistance of mouse double minute 2 homolog (MDM2), GTSE1 is able to transport p53 from the nucleus to the cytoplasm and promote its ubiquitination and degradation, thus affecting cell cycle and cell death-related signaling pathways. This paper reviews the expression of GTSE1 in lung cancer cells and its effects on lung cancer, as well as its potential mechanisms involved in cell cycle regulation.

肺癌是全球发病率和死亡率最高的恶性肿瘤^[1]^。化疗是治疗肺癌的主要手段之一，尤其对于老年晚期非小细胞肺癌（non-small cell lung cancer, NSCLC）患者^[2]^。然而，晚期肺癌患者往往会出现转移、复发、并发症和耐药性等问题，这些问题突显了提高药物抗肿瘤毒性在临床治疗中的重要性^[3]^。

细胞周期是细胞增殖的关键过程，涵盖了从生长到分裂的各个阶段^[4]^。肺癌细胞常见的异常包括细胞周期蛋白表达增加和细胞周期检查点功能失调，这些异常促进了细胞的无序增殖和肿瘤的形成^[5]^。紫杉醇、维帕莫司、顺铂等肺癌治疗常用药物与细胞周期紧密相关，不同药物在细胞周期的不同阶段发挥作用，因此，细胞周期的调控对于肺癌治疗至关重要。

G_2_和S期表达-1（G_2_ and S-phase expressed 1, GTSE1），也称为B99，是位于染色体22q13.2-q13.3的蛋白编码基因^[6]^。研究^[7⇓-9]^发现，GTSE1在细胞周期的DNA合成期及其后期的表达显著增加，这表明它可能参与了有丝分裂和DNA合成等细胞周期的关键生物学过程。大量研究^[10⇓⇓-13]^表明，GTSE1在骨肉瘤、前列腺癌、乳腺癌、肺癌等多种癌细胞中的表达显著升高。GTSE1不仅影响癌细胞的增殖、迁移和侵袭能力，而且作为一个重要的细胞周期调控蛋白，在细胞周期的调控中发挥着关键作用^[10⇓⇓-13]^。本文将深入探讨GTSE1与肺癌细胞周期之间的关系，以及它在肺癌细胞周期调控中的潜在作用，以期为肺癌治疗提供新的思路。

## 1 细胞周期及其调节

### 1.1 细胞周期的阶段

细胞周期是细胞生长和分裂过程中必须经历的生理阶段，包括四个主要阶段^[14]^。

Gap 1期（G_1_期）：是细胞周期的起始阶段。在此阶段，细胞不仅进行正常的生长和代谢活动，还会增加细胞质、细胞器和细胞周期调控蛋白等^[15]^。G_1_期中包含一个关键的决策点，称为“R点”（resting point）或“限制点”，在此细胞会检查DNA的完整性，评估细胞的健康状态和营养供应，确保没有严重损伤或错误，从而保证DNA能够正确复制，并决定是否进入下一个细胞周期阶段^[16]^。

Synthesis期（S期）：此阶段的主要任务是复制细胞的DNA。S期中，大量DNA聚合酶参与新DNA链的合成，并与原有DNA链互补配对，复制出两份相同的遗传信息，以确保在细胞分裂时每个新细胞都获得完整的遗传信息^[17]^。S期还设有检查点，以确保DNA复制的准确性和完整性。如遇DNA损伤或错误，细胞会暂停复制并启动修复机制，如同源重组修复或非同源末端连接^[18]^。若无法修复，或细胞无法正常进入细胞周期，将激活细胞凋亡，以维持基因组稳定性，防止错误的遗传信息传递给后代细胞。

Gap 2期（G_2_期）：作为第三个阶段，G_2_期的主要作用是为细胞分裂做准备。细胞在此期间合成和积累所需蛋白质和细胞器，如微管、中心体和蛋白质酶等，以支持分裂过程^[19]^。同时，细胞调控RNA及其他核酸的合成，确保M期时有足够的RNA等分子支持分裂。G_2_期也设有检查点，以确保DNA复制已完成且无损伤^[20]^。

Mitosis期（M期）：细胞周期的最后阶段，也是细胞分裂的关键阶段。M期负责将一个母细胞分裂成两个遗传信息完全相同的子细胞，分裂过程进一步细分为有丝分裂前期、中期、后期和末期^[21]^。如染色体分离或细胞分裂过程中出现问题，M期检查点机制将暂停分裂并进行修复或诱导细胞凋亡，以避免染色体不平等分配和其他潜在问题^[22]^。

### 1.2 细胞周期调控因子

细胞周期的调控依赖一系列蛋白质及其相关蛋白激酶，主要包括细胞周期蛋白（Cyclins）家族和细胞周期蛋白依赖性激酶（cyclin-dependent protein kinases, CDKs）。这些因子在细胞周期的各个阶段扮演至关重要的角色，确保细胞在适当的时机完成DNA复制和分裂^[23]^。Cyclins是一类在特定细胞周期阶段表达且在特定时间点降解的蛋白质。CDKs含有活性位点，能够与Cyclins结合，形成活性的Cyclin-CDK复合物。这些复合物通过磷酸化下游靶蛋白，对细胞周期进程进行关键调控^[24]^。例如，G_1_期的Cyclin D和Cyclin E与CDK4、CDK6和CDK2结合，推动细胞进入S期^[25]^；S期的Cyclin A与CDK2结合，促进DNA复制和细胞生长；G_2_期则受CDK1、Cyclin B、Wee1等蛋白质和激酶的调控，确保细胞有丝分裂在适当的时间和条件下进行，Cyclin B与CDK1的结合引导细胞进入有丝分裂^[26]^。

细胞周期蛋白相关激酶抑制蛋白（cyclin-dependent kinases inhibitors, CKIs）通过抑制CDKs的活性来调控细胞周期，影响细胞的DNA复制或分裂^[27]^。CKIs包括周期蛋白依赖激酶抑制因子1A（cyclin-dependent kinase inhibitor 1A, p21）、周期蛋白依赖激酶抑制因子1B（cyclin-dependent kinase inhibitor 1B, p27）和多肿瘤抑制基因（multiple tumor suppressor 1, p16）等。研究^[28]^表明，p21能够抑制多种CDK/Cyclin复合物的活性，阻止细胞从G_1_期进入S期。p27主要在细胞周期的G_1_/S和G_2_/M转换阶段抑制CDK2/Cyclin E和CDK2/Cyclin A复合物的活性，调节细胞的DNA复制和有丝分裂^[29]^。p16作为一种抑癌基因产物，在G_1_期通过抑制CDK4/Cyclin D和CDK6/Cyclin D复合物的活性，阻止细胞从G_1_期进入S期（图1）^[30]^。

**图1:**
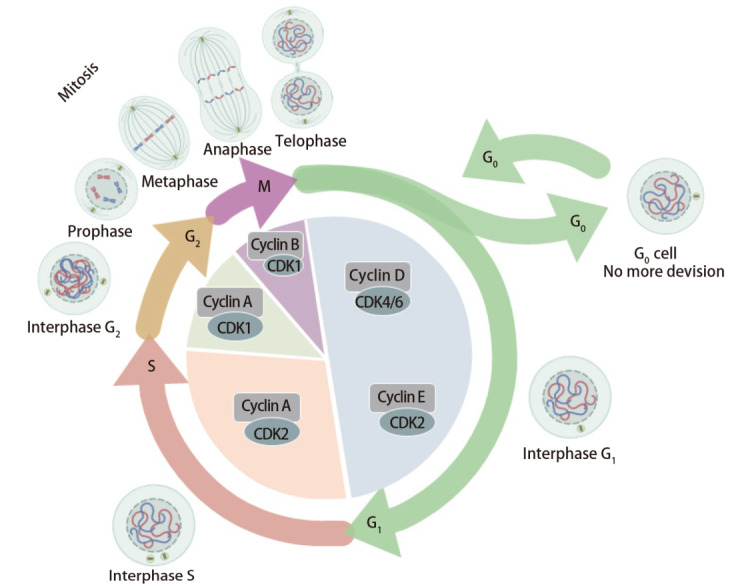
细胞周期及细胞周期调节因子

### 1.3 细胞周期调节异常与肺癌的发生和发展

细胞周期调节异常是导致肺癌过度增殖的关键因素之一。正常情况下，细胞周期能够精确控制细胞的生长和分裂。然而，当细胞周期调节失常时，细胞可能进入异常的增殖状态，导致过度增殖^[31]^。例如，G_1_/S过渡期的调控失调会使细胞过早进入DNA复制阶段，从而促进肺癌细胞的形成^[32]^。此外，细胞周期调节异常还会促进肿瘤血管生成，为肿瘤提供足够的氧气和营养，并为肿瘤细胞的转移创造条件^[33]^。

细胞周期调节异常还影响肺癌细胞对DNA损伤的敏感性。通常情况下，当肺癌细胞受到DNA损伤时，细胞周期会停滞并启动DNA损伤应答反应。如果修复失败，细胞将诱导凋亡。然而，当细胞周期调节异常时，不仅会影响细胞对DNA损伤的应答，还会抑制细胞凋亡功能，使肺癌细胞能够逃脱凋亡信号的识别和执行^[34]^。研究^[35]^指出，细胞周期调节蛋白与凋亡相关因子之间存在复杂的交互作用，例如，肿瘤蛋白53（tumor protein 53, p53）是一个关键的细胞周期调节蛋白，同时也是一个重要的凋亡调节因子。在细胞DNA受到损伤时，p53能够调控细胞周期的停滞以修复DNA或诱导凋亡，并影响凋亡相关蛋白家族成员的表达^[36]^。当细胞周期调节失调时，这些交互作用可能受到干扰，从而阻碍肺癌细胞正常传递凋亡信号。

## 2 GTSE1的功能

GTSE1目前在多种人类癌症中被证实为致癌基因，其编码的蛋白分子包含大约650个氨基酸残基，且在不同组织和细胞类型中广泛表达^[37]^。GTSE1功能域包括富含丝氨酸和苏氨酸的突触后密度蛋白95/大盘基因/闭锁小带蛋白1结构域（post-synaptic density protein 95/discs large homolog/zonula occludens-1, PDZ）等多个蛋白质相互作用结构域，这使得GTSE1能够与多种蛋白质相互作用，并参与调节细胞的各种生理过程^[38]^。

### 2.1 GTSE1在细胞周期调控中的作用

GTSE1是细胞周期调控中的关键分子，特别是在G_2_和S期，其表达水平显著升高。研究^[39]^发现，GTSE1在多种细胞类型中都能调控细胞周期。Zheng等^[40]^在肝癌细胞中观察到，GTSE1通过激活细胞周期，加剧了肝癌的不良预后。Guo等^[41]^的研究表明，在肝癌细胞中抑制GTSE1的表达，可降低G_2_/M期关键调节蛋白Cyclin B1的水平。Lin等^[42]^在乳腺癌细胞中发现，GTSE1能够影响p53的功能，对依赖p53的细胞周期调控产生影响。Bublik等^[43]^在对人骨肉瘤的研究中发现，GTSE1的N端部分能够与p21结合，使其稳定并避免降解，由于p21能通过与CDK-Cyclin复合物和增殖细胞核抗原结合来阻止细胞周期的进展，因此他们认为，通过稳定GTSE1的表达，可以有效防止p21的降解，从而稳定细胞周期。以上研究表明，GTSE1通过与不同细胞周期蛋白相互作用，发挥着调控细胞周期进程的重要作用。

研究^[44]^表明，GTSE1与细胞周期蛋白依赖性激酶的CDK1和CDK2之间存在密切关系。在黑色素瘤中GTSE1与CDK1结合后可抑制CDK1与其配体Cyclin B形成活性的CDK1/Cyclin B复合物，从而阻止细胞进入有丝分裂，实现G_2_/M期的阻滞^[45]^。此外，GTSE1还可与Cyclin B1结合，这种相互作用有助于调控细胞进入有丝分裂期，影响有丝分裂的进行^[46]^。在宫颈癌中GTSE1的表达与Cyclin D1和Cyclin E的表达呈正相关，且GTSE1可以结合CDK4，影响G_1_/S期的过渡^[47]^。综上所述，GTSE1通过与Cyclins、CDKs相互作用，参与调节细胞周期的不同阶段。

### 2.2 GTSE1在微管结构调控中的作用

GTSE1在细胞中对微管结构具有重要作用。Wang等^[48]^发现，在NSCLC细胞中GTSE1影响Tau蛋白、Stathmin1蛋白和微管相关蛋白的表达；此外，GTSE1低表达会导致微管结构紊乱。Bendre等^[49]^观察到，在细胞有丝分裂过程中，GTSE1定位于中心粒区域和微管网中，通过与α-β微管动力蛋白相互作用，GTSE1获得精细的微管调控功能，从而在维持细胞骨架和有丝分裂的正常进程中发挥关键作用。

Singh等研究^[9]^发现，通过调控CDK1的活性，GTSE1可以从长星体微管的正端被移除，这一过程不仅影响了微管的稳定性，也进一步影响了细胞的增殖能力。此外，Xklp2靶向蛋白（targeting protein for Xklp2, TPX2）和Aurora激酶家族成员作为纺锤体蛋白，在纺锤体的形成和功能中发挥至关重要的作用^[50]^。Yao等^[51]^在透明细胞肾细胞癌的研究中发现，随着细胞进入有丝分裂前期，GTSE1的表达水平发生变化，并且与TPX2的相互作用促进了纺锤体的形成和结构稳定性。GTSE1还通过影响Aurora激酶家族成员的磷酸化，调节纺锤体微管的动力学和结构^[52]^。以上发现表明，GTSE1不仅参与调控细胞的微管结构以维持细胞骨架的稳定，同时在细胞有丝分裂过程中还能够确保染色体的准确分离。

### 2.3 GTSE1在细胞凋亡调控中的作用

GTSE1作为细胞凋亡调控的重要因子，其表达水平与细胞的凋亡抵抗和敏感性密切相关。研究表明，GTSE1的过度表达可能导致肿瘤细胞的凋亡抵抗。例如，在结直肠癌细胞中，GTSE1过度表达使得这些细胞对化疗药物5-氟尿嘧啶的敏感性降低，并且与凋亡信号的抑制有关^[53]^。另一方面，在胃癌中，抑制GTSE1的表达能够增加胃癌细胞对化疗药物顺铂的敏感性，促进细胞的凋亡^[54]^。GTSE1与凋亡相关蛋白p53的相互作用是其调控细胞周期的重要机制之一^[55]^。通过将p53从细胞核转位到细胞质中，并与泛素连接酶MDM2结合，GTSE1促使p53蛋白泛素化，从而影响p53的转录活性和细胞凋亡信号通路的激活，抑制细胞凋亡^[56]^。此外，还有研究^[57]^发现GTSE1与B淋巴细胞瘤-2基因（B-cell lymphoma-2, Bcl-2）家族中促进凋亡蛋白具有相互作用，影响线粒体途径中的凋亡信号传导。综上所述，GTSE1在细胞凋亡调控中扮演着关键角色。

## 3 肺癌中GTSE1的表达及其对肺癌的影响

Kaushik等^[58]^研究发现，相对于配对的正常肺组织，肺癌组织中GTSE1的表达水平显著上调（P<0.05）。Wang等^[48]^也对36例配对组织进行了GTSE1表达验证，发现有24例组织样本的GTSE1表达明显高于配对组织样本。此外，与人正常肺支气管上皮细胞（Beas-2b细胞）对比，肺癌细胞系（H146、H82、H460、A549）中GTSE1的mRNA水平也显著上调^[59]^。

综合来自癌症基因组图谱（The Cancer Genome Atlas, TCGA）、基因表达综合数据库（Gene Expression Omnibus, GEO）和欧洲基因组-表型档案（European Genome-phenome Archive, EGA）数据库的1926例NSCLC病例分析研究发现GTSE1的高表达与NSCLC患者较短的总生存期显著相关（P<0.01）^[58]^。此外，对NSCLC患者的病理参数进行整理分析，结果表明GTSE1高表达患者比GTSE1低表达患者有更高的淋巴结转移概率^[60]^。通过MetaDE.ES分析、构建预后相关基因风险评分模型、Kaplan-Meier生存分析等方法，Jin、Zhang等^[61,62]^研究者也发现GTSE1的表达水平与肺癌患者的预后显著相关。Han等^[63]^整合GEO数据库中的四个基因表达谱，并进行了基因本体分析，结果显示GTSE1的表达上调与肺腺癌和肺鳞状细胞癌的不良预后有密切关系，这表明GTSE1在肺癌的诊断和治疗中可能具有潜在的应用价值。

Tan等^[64]^研究发现，GTSE1可能抑制肺癌的免疫反应，通过计算泛癌患者的基质评分和免疫评分，发现肺癌的基质评分和免疫评分呈负相关。Lei等^[65]^的实验表明，当抑制NSCLC细胞系（A549、H460和H1299）中的GTSE1表达后，这些细胞对放射治疗的敏感性明显增强，这一现象与DNA损伤标志物磷酸化组蛋白H2A变体X（phosphorylated histone h2A variant X, γH2AX）的增加有关。此外，细胞周期检查点在响应DNA损伤时发挥着重要作用，GTSE1的调控可能影响这一关键细胞的应答机制^[66]^。

目前关于GTSE1表达对肺癌化疗治疗的影响尚无明确的研究。然而，有一些研究值得关注。在肝癌中GTSE1与上皮间充质转化调节的迁移和侵袭密切相关。此外，GTSE1还可促进透明细胞肾细胞癌的细胞增殖、细胞周期转变、迁移和侵袭能力^[56,67]^。在肺癌中，GTSE1高表达与细胞的增殖、迁移、侵袭有关^[48,59]^。早期研究^[68]^表明，细胞周期蛋白如Cyclin D和CDK4/6可以调节细胞迁移和侵袭相关基因的表达，从而影响细胞迁移和侵袭的发生。Zhang等^[59]^的实验结果显示，抑制A549、H460细胞系中的GTSE1表达导致细胞周期发生明显改变，包括G_1_期阻滞和G_2_期减少，同时p53蛋白明显增加。

虽然目前关于GTSE1对肺癌细胞周期调控的机制研究还处于初期，但这些发现表明GTSE1在肺癌中可能通过调控细胞周期影响细胞功能。

## 4 GTSE1在肺癌细胞周期调控中的潜在机制

Monte等^[8]^研究发现，GTSE1与p53之间的相互作用是肺癌研究中的一个重要研究方向，他们发现p53的C端结构域与GTSE1的C端区域之间存在物理相互作用。进一步研究^[69]^表明，p53能够通过与p21上的p53响应元件结合，形成一个复合物，并招募其他转录因子，促使RNA聚合酶II结合并启动p21的转录，p21则通过降低Cyclin-CDK复合物的活性，抑制细胞周期的进展。基于这些发现，我们认为未来的研究应该深入探究肺癌细胞中GTSE1与p21表达之间的关系，并阐明GTSE1如何调控细胞周期分布的具体机制，这将有助于更好地理解GTSE1在肺癌发展中的作用，并可能为治疗提供新的靶点。

Lei等^[65]^的研究发现，当肺癌细胞DNA受损后，GTSE1会移动到受损位置并促进DNA损伤的修复，抑制GTSE1表达后，DNA修复功能降低。此外，其他研究^[70]^表明，DNA损伤信号通路中的共济失调-毛细血管扩张突变蛋白（ataxia-telangiectasia mutated, ATM）或共济失调和辐射敏感蛋白3相关蛋白（ATM and Rad3-related, ATR）能够磷酸化p53，使其从不稳定的形式转变为稳定且活性的形式，从而诱导细胞凋亡，防止受损细胞异常增殖。基于这些发现，我们认为抑制肺癌细胞中GTSE1的表达可能促进p53的增加，或者有助于解决DNA损伤药物顺铂在临床应用中的耐药问题。

Zhang等^[59]^研究发现，GTSE1在肺癌细胞系（H460和A549细胞）中能够激活蛋白激酶B（protein kinase B, AKT）/哺乳动物雷帕霉素靶蛋白（mammalian target of rapamycin, mTOR）信号通路，这一发现对于肺癌细胞的增殖、迁移和侵袭以及细胞凋亡的抑制具有重要意义。AKT/mTOR信号通路在细胞周期蛋白的调控中也起着关键作用。在肺癌细胞中，活化的AKT可以通过直接磷酸化作用或通过mTORC1通路影响S6激酶1（ribosomal protein S6 kinase beta-1, S6K1）和4E结合蛋白1（eIF4E-binding protein 1, 4E-BP1），从而间接调控细胞周期^[71,72]^。AKT的活性不仅可以抑制糖原合成酶激酶-3，还可以减少p21和p27的降解，实现对G_1_/S期的过渡调控^[73]^。此外，AKT还能够调控叉头盒O转录因子（forkhead box O, FOXO）家族成员的表达，影响细胞周期蛋白的核转位。FOXO蛋白的活性会导致癌细胞发生G_1_期阻滞，而其被AKT磷酸化后的核排出则促进了细胞进入S期^[74]^。S6K1通过磷酸化小核糖体亚基中的S6蛋白，促进Cyclin D1等细胞周期蛋白的合成^[75]^。mTOR1通过抑制真核翻译起始因子4E结合蛋白1（eukaryotic initiation factor 4E-binding protein 1, 4E-BP1）活性，促使Cyclin D1、Cyclin E等细胞周期蛋白的合成^[76]^。通过深入探讨GTSE1对AKT/mTOR信号通路下游因子的影响，可以进一步阐明GTSE1如何调控肺癌细胞周期，这为肺癌的诊断和治疗提供了新的思路。

## 5 结论

综上所述，GTSE1在肺癌细胞周期调节中发挥着重要作用，通过干预GTSE1与细胞周期调节蛋白的相互作用，或者调节其表达水平，可以影响p53/p21、AKT/mTOR等细胞周期相关的信号通路，从而抑制肺癌细胞的增殖、迁移、侵袭能力。GTSE1与细胞周期的紧密关联使其成为肺癌研究和治疗的潜在靶点和标志物，深入研究GTSE1与肺癌细胞周期之间的关系，有助于探讨肺癌发生和发展的相关机制。

尽管GTSE1对于细胞周期有着显著的影响，但目前研究揭示出在不同细胞类型中，调控细胞周期的方式或结果可能存在差异。例如，GTSE1本身可以结合p21并保护p21免受降解，在p53突变型或p53缺失型细胞中，沉默GTSE1会导致p21表达下降。然而，在p53野生型细胞中，沉默GTSE1能够抑制p53的降解，从而促进p21转录。未来在肺癌诊治中，如何利用GTSE1调控细胞周期配合临床一线治疗药物的使用还需要进一步探究。
